# Geographical Landscape and Transmission Dynamics of SARS-CoV-2 Variants Across India: A Longitudinal Perspective

**DOI:** 10.3389/fgene.2021.753648

**Published:** 2021-12-17

**Authors:** Neha Jha, Dwight Hall, Akshay Kanakan, Priyanka Mehta, Ranjeet Maurya, Quoseena Mir, Hunter Mathias Gill, Sarath Chandra Janga, Rajesh Pandey

**Affiliations:** ^1^ Integrative Genomics of Host-Pathogen (INGEN-HOPE) Laboratory, CSIR-Institute of Genomics and Integrative Biology (CSIR-IGIB), Delhi, India; ^2^ Department of Biohealth Informatics, School of Informatics and Computing, Indiana University Purdue University, Indianapolis, IN, United States; ^3^ Academy of Scientific and Innovative Research (AcSIR), Ghaziabad, India

**Keywords:** COVID-19, VOCs, genomic surveillance, transmission dynamics, integrative analysis, longitudinal, Auspice, Nextstrain

## Abstract

Globally, SARS-CoV-2 has moved from one tide to another with ebbs in between. Genomic surveillance has greatly aided the detection and tracking of the virus and the identification of the variants of concern (VOC). The knowledge and understanding from genomic surveillance is important for a populous country like India for public health and healthcare officials for advance planning. An integrative analysis of the publicly available datasets in GISAID from India reveals the differential distribution of clades, lineages, gender, and age over a year (Apr 2020–Mar 2021). The significant insights include the early evidence towards B.1.617 and B.1.1.7 lineages in the specific states of India. Pan-India longitudinal data highlighted that B.1.36* was the predominant clade in India until January–February 2021 after which it has gradually been replaced by the B.1.617.1 lineage, from December 2020 onward. Regional analysis of the spread of SARS-CoV-2 indicated that B.1.617.3 was first seen in India in the month of October in the state of Maharashtra, while the now most prevalent strain B.1.617.2 was first seen in Bihar and subsequently spread to the states of Maharashtra, Gujarat, and West Bengal. To enable a real time understanding of the transmission and evolution of the SARS-CoV-2 genomes, we built a transmission map available on https://covid19-indiana.soic.iupui.edu/India/EmergingLineages/April2020/to/March2021. Based on our analysis, the rate estimate for divergence in our dataset was 9.48 e-4 substitutions per site/year for SARS-CoV-2. This would enable pandemic preparedness with the addition of future sequencing data from India available in the public repositories for tracking and monitoring the VOCs and variants of interest (VOI). This would help aid decision making from the public health perspective.

## Background

RNA viruses such as SARS-CoV-2 exist as a swarm of genetically related variants and not as a single genotype. This property enables the virus to change hosts and adapt to changing environmental conditions. This is achieved through polymerase fidelity and genomic recombination that regulate this feature of RNA viruses (Barr and Fearns, 2016). In a pandemic, these features lead to generation of strains with modified epidemiological characteristics such as changing transmissibility, virulence, and varying immunological characteristics leading to altered vaccine, drug, and detection efficacy (Davies et al., 2021a; Becker et al., 2021; U.S, Food and Grug Administration, 2021). Based on these characteristics, WHO has classified SARS-CoV-2 variants as variants of concern (VOC) and variants of interest (VOI), achieved through global genomic surveillance efforts. The VOC—alpha, beta, gamma, and delta—have been shown to have increased transmissibility or virulence and suggested to have increased immune evasion (WHO., 2021).

The first designated VOC is the alpha variant with the prominent mutations, N501Y and 69-70del. Compared to the wild type Wuhan strain, this variant has been shown to have properties of increased transmission, increased mortality rate, and reduced efficiency of RT-PCR–based detection (Davies et al., 2021a; 2021b; U.S, Food and Grug Administration, 2021). Subsequently, variants of beta with characteristic mutations K417N and E484K, gamma with K417T, and delta with mutations L452R, T478K, and P681R have been designated as VOC (Supplementary Table S1). The VOC delta (B.1.617.2) has been identified as the major strain during the second COVID-19 surge in India. The variant has been reported to have immune evasion and higher transmission characteristics (Dhar et al., 2021; Wall et al., 2021). Studies from India have elucidated the presence of different strains in specific parts/states of the country such as West Bengal (Begum et al., 2020) having the B.1 lineage in April 2020, Kerala (Radhakrishnan et al., 2021) with the B.1 lineage in August 2020, Telangana (Gupta et al., 2021) having the B.1.1.25 lineage in the period April–July 2020, and Gujarat (Joshi et al., 2021) observed to have B.1.36 and B.1 lineages in April 2020. Such real-time reporting of the findings from different research groups across India have been useful to highlight the spectrum of SARS-CoV-2 lineages and the evolution of their mutations. However, most of these current efforts have not provided a comprehensive view of the transmission and evolutionary dynamics from a pan-India perspective using thousands of SARS-CoV-2 genomes from India. Such pan-India efforts are especially important during a pandemic to understand the emergence, spread, and evolution across timescales for populous countries which would otherwise be missed, albeit unintentionally, from region-/cohort-specific study/ies (Hassan et al., 2020; Kumar et al., 2020; Sarkar et al., 2021). Toward this, recent initiatives such as Indian SARS-CoV-2 Genomic Consortia (INSACOG), with a pan-India geographic footprint, have been extremely useful. With the global sharing of sequences from different parts of India to global repositories like GISAID, it provides an opportunity towards a granular view of SARS-CoV-2 evolution. While we track the emergence of specific variants to specific time points in the pandemic, a longitudinal view of the virus vis-à-vis India has been lacking. In this study, we addressed several of these limitations from smaller scale studies focusing on Indian SARS-CoV-2 genomes in recent times, including i) pan-India representation, ii) longitudinal view from April 2020–March 2021, iii) SARS-CoV-2 genome sequences deposited in GISAID, iv) more than 99% genome coverage, v) sample metadata, vi) nucleotide substitution rate, and vii) transmission dynamics within India. The comprehensive data analysis with in-depth phylogeny, lineage, co-analysis, and an interactive visualization tool by Nextstrain, adds to the strength of the study (Hadfield et al., 2018). This not only boosts our insights into viral evolution but also presents a prospective publicly accessible platform for customizable visualization of SARS-CoV-2 genomic data across India. The dashboard would augment other existing ones, inclusive of https://nextstrain.org/community/banijolly/Phylovis/COVID-India, with an aim to strengthen the SARS-CoV-2 genome surveillance and information dissemination. With certain unique and overlapping strengths, in combination, they would be helping to achieve the common goal of a better and efficient genetic epidemiological surveillance.

## Results

### COVID-19 Genomic Data Characteristics

**FIGURE 1:**
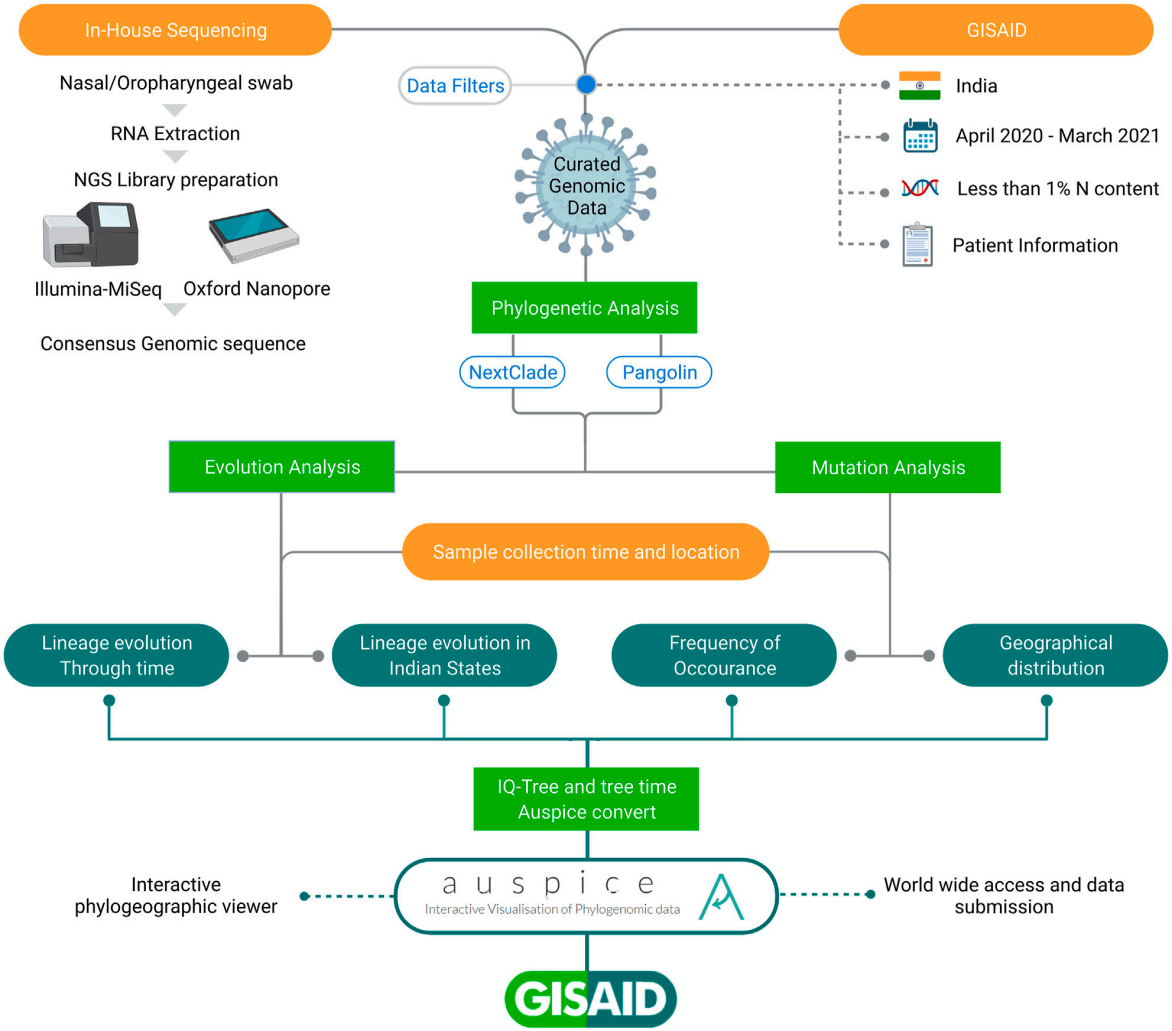
Schematic representation of workflow adopted in the study. All the SARS-CoV-2 sequences have been taken from GISAID inclusive of in-house and those submitted by others from India. The integrative data analysis for the longitudinal time period highlights lineage evolution through time, in different Indian states and Union Territories, mutation frequencies, and their geographical distribution.

We generated 1144 SARS-CoV-2 genomic sequences using ONT (*n* = 927) and Illumina MiSeq (*n* = 217) platforms *via* in-house sequencing of COVID-19 positive samples, as detailed in the methods section. We also downloaded 9618 Indian SARS-CoV-2 genome sequences from GISAID. After merging and applying sequencing quality filters, we retrieved a total of 10,183 sequences. Then, by filtering out sequences related to patients’ metadata lacking date of sample collection, sampling location, age, and gender information, we retrieved a final set of 7631 sequences used for analysis presented in this study and Auspice data visualization. The data were segregated according to the location information for the 28 Indian states and union territories (UTs) with age and gender information across the time period of 12 months (April 2020–March 2021). These curated data were used in this study, as highlighted in the workflow shown in Figure 1.

### Demographics and Phylogeography of SARS-CoV-2 Genomes in India

**FIGURE 2:**
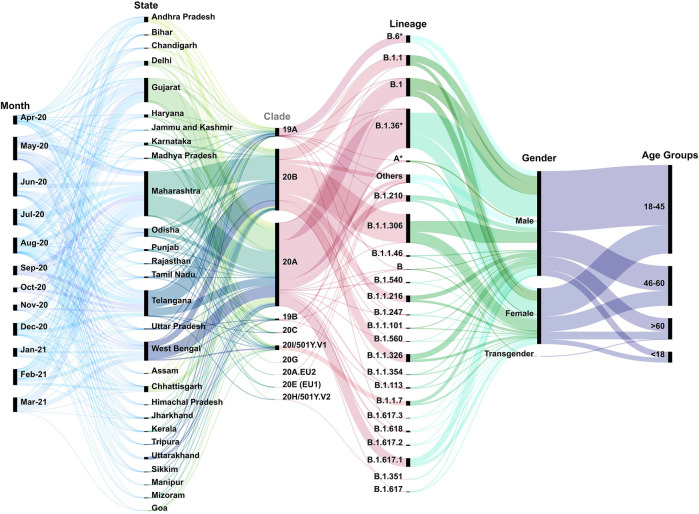
Multi-categorical statistical plot threading the variables longitudinally. The figure has captured the variables of time, states/UTs, clades, lineages, gender, and age of the patients across India from the month of April 2020–March 2021. It highlights the diversity of the above variables as well as their inter-relatedness. The width of bands indicates the number of samples pertaining to the specific criteria.

Upon phylogeographic and demographic segregation, we observed clades 20A and 20B to be predominant in most of the Indian states/UTs between the months of April 2020 and March 2021 (Figure 2). It is important to mention that there is inequality of sequencing data from different parts of India over the time period of this study. We noticed a relatively lower number of sequences from the states of Bihar, Punjab, Madhya Pradesh, Uttar Pradesh, and Rajasthan. At the same time, a higher percentage of the sequencing data has been generated from Maharashtra 28.42% (*n* = 2,168), Telangana 15.03% (*n* = 1,147), West Bengal 11.58% (*n* = 884), Gujarat 14.78% (*n* = 1,128), and Delhi 2.72% (*n* = 208). The longitudinal data analysis provides insights into the sequences being generated and deposited across the months in addition to the different states/UTs. We observed a drop in sequencing in the months of Oct-Nov 2020. This is overlapping with lower COVID-19 cases in India during the time period. In the analyzed data, we observed a gender representation of 2:1 males: females which is possibly a reflection of the working population and exposure to the SARS-CoV-2 infection. We also found that the working class age group of 18–45 has a higher representation which accounts for 51.19% (*n* = 3,907) of the total samples.

Additionally, we observed a spectrum of lineages across the time period in different states and UTs of India. This included B.1 (11.19%), B.1.1 (6. 97%), B.1.1.306 (17.10%), B.1.1.326 (4. 70%), B.1.1.7 (2.98%), B.1.36* (B.1.36 and its sub-lineages) (24.99%), B.1.617.1 (6%), and B.6* (B.6 and its sub-lineages) (4.33%) in the dataset. In this study, we have aggregated the others to include those lineages which have frequency less than 0.34 (Supplementary Table S2).

### Evolution of SARS-CoV-2 Phylogeny in India

**FIGURE 3:**
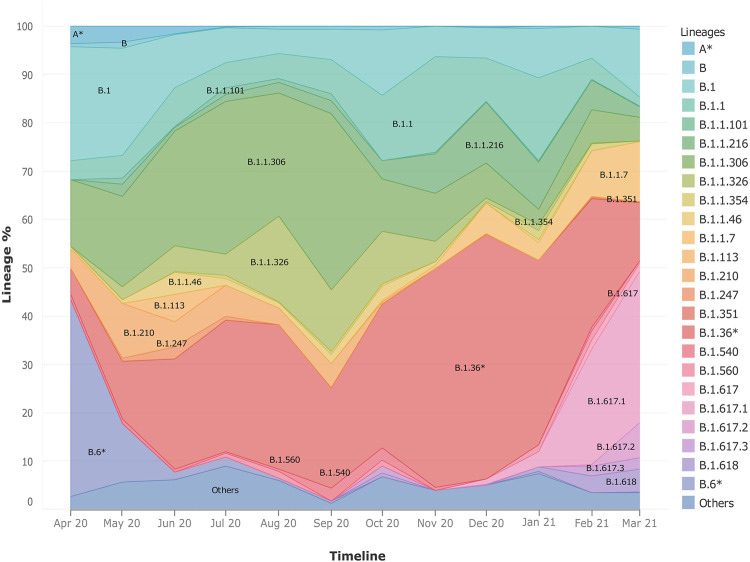
Frequency graph showing longitudinal distribution of lineages. It highlights the pan-India distribution of lineages with respect to time in India during the months of April 2020–March 2021. It also helps to highlight the dynamic presence of lineages in time and space.

Upon observing the lineage distribution across the time span of April 2020–March 2021, we observed few striking trends (Figure 3). We discovered the presence and subsequent growth of the B.1.617 lineage and its sub-lineages from October 2020 onward. Pan-India longitudinal data also highlighted that B.1.36* was the predominant clade in India until Jan-Feb 2021 after which it has gradually been replaced by the B.1.617.1 lineage, from December 2020 onward. We also observed a steady growth of the B.1.1.7 lineage since its first detection in the month of October 2020. The lineages B.1.1.306 and B.6*, which used to be the predominant lineage in India during the initial phase of the COVID-19 pandemic, were found to have been replaced by other national and international strains.

**FIGURE 4:**
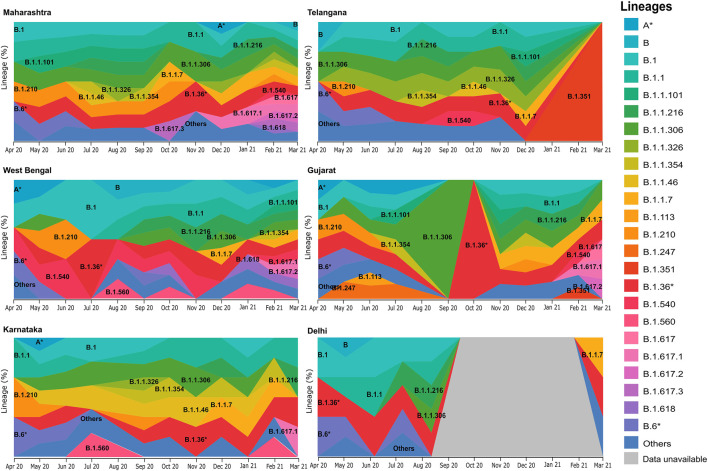
Frequency graph distribution of lineages in specific states/UTs. This captures the differential distribution of lineages with respect to time in specific Indian states/UTs with relatively high sequencing data during the 12-month period of April 2020–March 2021.

Upon distribution of country wide phylogenetic data into respective states, we are able to compare the national and local trends in virus propagation (Figure 4). Subsequently, we zoomed into specific states and UTs for a detailed understanding of the contribution of the strains and their spread. In the state of Maharashtra, we observed that the B.1.617* clade emerged around October 2020, and over the next few months, it replaced other previously predominant clades. Our analysis highlighted that B.1.617.3 was first seen in India in the month of October in the state of Maharashtra. The now most prevalent strain, B.1.617.2, was first seen in Bihar based on the same metadata and subsequently, (Supplementary figure S1) in the states of Maharashtra, Gujarat, and West Bengal. Contrasting to the national trend, in Telangana, we see a major rise of B.1.351. The lineage B.1.618 was first seen in West Bengal in the month of October 2020 before it spread to Chhattisgarh, Delhi, and Maharashtra.

### Diversity of Genetic Variants of SARS-CoV-2 in Different Geographic Regions of India

Throughout a pandemic, the accumulation of mutations within the genome is the fundamental driving force behind viral evolution. Therefore, we carried out a variant analysis of circulating SARS-CoV-2 genomes to understand the abundance of mutations with respect to the geographical distribution within India. Analysis revealed the occurrence of 13,184 different unique mutations in the 7631 SARS-CoV-2 genomes. When mapped with respect to state-wise distribution with relatively higher sequence coverage, a total of 2093 mutations in Delhi, 5254 in Gujarat, 2445 in Karnataka, 8396 in Maharashtra, 3990 in Telangana, and 4244 mutations in West Bengal were detected in the SARS-CoV-2 sequences. These are non-unique sets of mutations distributed across the states/UTs.

**FIGURE 5:**
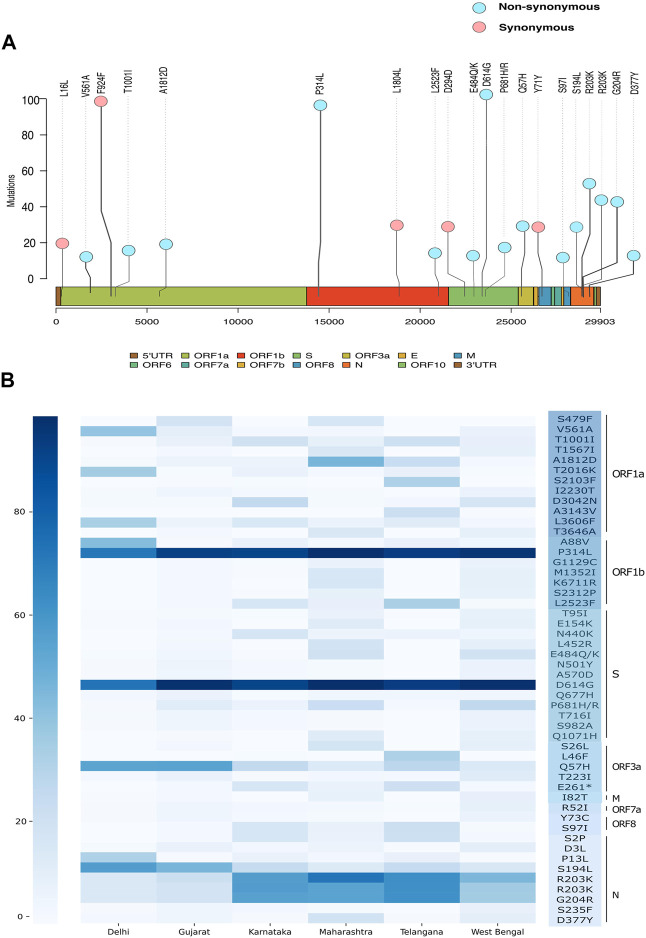
Genetic mutation composition of SARS-CoV-2 in India. **(A)** Most frequent mutations observed across Indian sequences. **(B)** Heatmap showing frequency distribution of top 50 non-synonymous mutations in Delhi, Gujarat, Karnataka, Maharashtra, Telangana, and West Bengal.

Upon observing the frequency of occurrence of each mutation (Supplementary Table S3), we see two non-synonymous mutations A23403G (S: D614G) and C14408T (ORF1b:P314L) and a synonymous mutation C3037T (ORF1a:F924), C241T (5′UTR) in more than 90% of all the viral genomes in our dataset (Figure 5A). These are signature mutations of the B.1 lineage and its derivative lineages that have spread early during the pandemic. These mutations are followed by non-synonymous mutations at G28881A (N: R203K), G28882A (N: R203K), and G28883C (N: G204R) which were found in >40% of the genomes representing the B.1.1 lineage. The defining mutations of the lineage B.1.617 (P681R, L452R), its sub-lineage B.1.617.1 (P681R, L452R, E154K, E484Q, and Q1071H), and B.1.1.7 (T1001I, I2230T, S235F, D3L, R52I, N501Y, P681H, and T716I) were also found frequently. Subsequently, we looked at the top 50 frequently occurring non-synonymous mutations across all states (Figure 5B, Supplementary Table S4). We found that the states of Gujarat, Maharashtra, Karnataka, and Telangana follow a similar pattern of non-synonymous mutations overall, whereas Delhi has a distinctive pattern. Apart from the D614G and P314L mutations, all five states share mutations at positions G204R, Q57H, R203K, and S194L in varying frequency. Mutations A88V, T2016K, and P13L were predominant in Delhi samples, while mutations L2523F, L46F, and S2103F were observed in the sequences from the state of Telangana.

### Divergence of the SARS-CoV-2 Genome Across India

**FIGURE 6:**
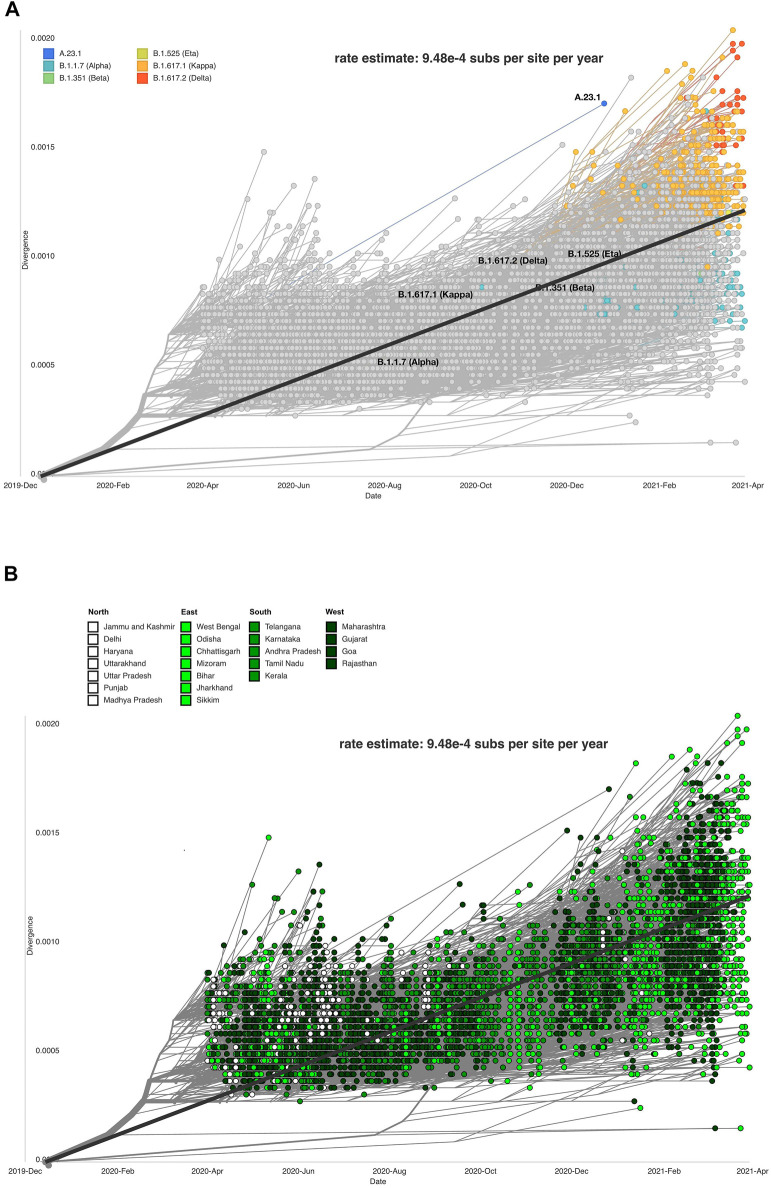
Data visualization using Auspice depicting SARS-CoV-2 genome divergence. **(A)** respective lineages (Auspice), and **(B)** respective Indian states (Auspice/India).

The evolutionary rate estimates the mutations within virus genome/s, which facilitates the understanding of how the virus will deviate from the original strain. The rate estimate for the divergence of our dataset is 9.48 e-4 substitutions per site per year or 28.44 nucleotide substitutions per year. Other studies have found SARS-CoV-2 to have an evolutionary rate of 8 × 10^–4^ substitutions per site/year (Duchêne et al., 2014; MacLean et al., 2020; Su et al., 2020).The accuracy of the mutation rate estimate of our data as compared to the global and theoretical mutation rate of the virus is in concurrence with the accuracy of divergence projections inferred from our dataset. Clade, lineage, and mutation information was processed by the Nextstrain system to produce a divergence plot with respect to lineages and clade distribution across Indian states/UTs (Figures 6A,B).

The sequences and states were divided into north, south, east, and west (Figure 6B). The purpose of this analysis was to study if certain parts of the country were exhibiting more divergence in the observed strains of SARS-CoV-2 than others. The eastern states showed the most variability on the divergent scale, but other regions also vary across the plot. Southern states clustered close to the regression line (intercept = −1. 28, slope = 0. 000634, R^2 = 0. 481) plotted on the figure. It is not conclusive which region has the most divergent sequences, but we observe that the SARS-CoV-2 genome sequences from the northern states of India gradually reduced in the months following October 2020.

### Hosting a Data Visualization System for Indian SARS-CoV-2 Genomic Data

**FIGURE 7:**
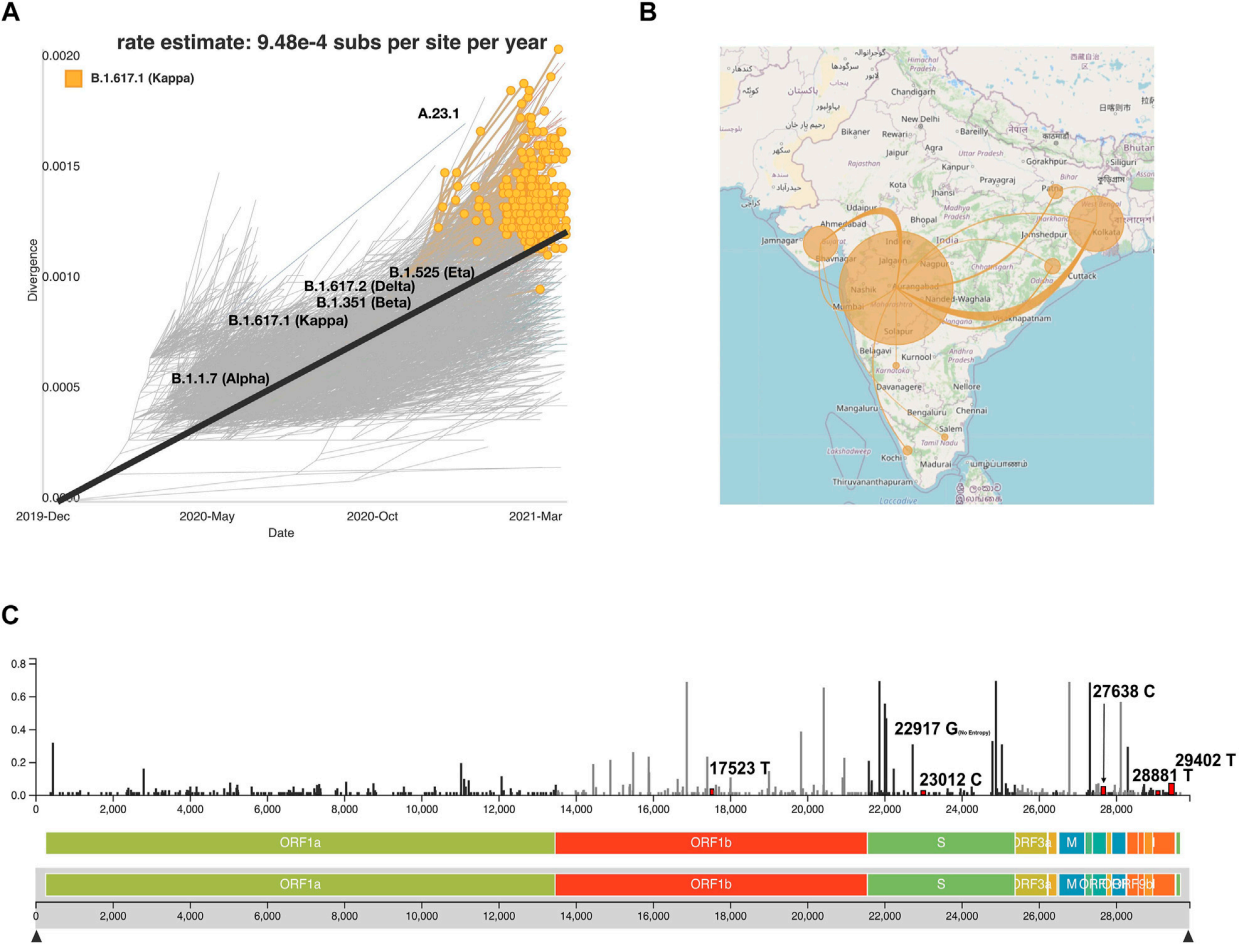
Auspice viewer displaying **(A)** the phylogenetic evolution for the lineage B.1.617.1. **(B)** Transmission lines displaying the locations most similar to the lineage B.1.617.1 (YouTube., 2021) and the transmission lines without the B.1.617.1 filter across Indian states (YouTube., 2021). **(C)** Genomic diversity chart displaying the nucleotide substitutions for B.1.617.1 (substitutions, 17523T, 22917G, 23012C, 27638C, 2881T, and 29402T). Nucleotide substitutions are highlighted in red and labeled next to their respective entropy levels.

To enable an understanding of the transmission and evolution of the SARS-CoV-2 genomes that were analyzed in this study, we built a transmission map to display transmission patterns of the phylogenetic analysis by utilizing the Nextstrain system by employing collection date, location, and sequence similarity data. This system along with the complete data employed in this study is available (auspice/India) along with documentation to implement it. The strength of the map includes its potential to find places with similar mutations, clades, and lineages. This interactive system can track the phylogenetic tree as highlighted in the example snapshot shown for lineage B.1.617.1, the most frequent lineage in our dataset (Figure 7A). Transmission lines are predicted for each location with similar sequences to the B.1.617.1 lineage (Figure 7B). Nextstrain allows for users to define lineages and clades. Figure 7C highlights a lollipop plot showing the nucleotide substitutions 17523T, 22917G, 23012C, 27638C, 2881T, and 29402T associated with the B.1.617.1 lineage. Users can select mutations with high entropy or mutation events, and the system enables tracking locations with similar mutations (Figure 7B). This tool is an innovative way to track the evolution of the SARS-CoV-2 virus (Hadfield et al., 2018; Huddleston et al., 2021). In order to systematically track the evolution of the SARS-CoV-2 genome, we plan to continue the addition of new SARS-CoV-2 genomes in regular intervals to the Nextstrain system. Such efforts would not only be extremely useful for scientists and public health experts to understand the transmission dynamics, mutational divergence, and geographical abundance of new and emerging variants but also aid the common public to understand the spread of infections across India aiding the relatively early detection of emerging VOCs.

## Discussion

Nearly 6 months after the peak of the first wave of COVID-19 in September 2020, coronavirus cases in India started rising alarmingly from the first week of March 2021 signaling the possible arrival of the second wave of the pandemic in the country. This necessitated localized lockdowns to contain spread and associated hospitalization of the patients with severe symptoms. With multiple SARS-CoV-2 milestones inclusive of the initial introduction of SARS-CoV-2 in India, re-infection cases, VOCs, second surge, and the vaccine breakthrough, it is probably important to integrate the horizontal and vertical views of the pandemic. Toward this, genomic surveillance has been integral with the data being submitted to global repositories, such as GISAID. The global data sharing has greatly facilitated national and global understanding toward patterns, evolution, and transmission dynamics of SARS-CoV-2. In the current study, we try to elucidate the sequencing demographics, transmission dynamics, lineage evolution, and geographical distribution of SARS-CoV-2 variants across India by integrating the SARS-CoV-2 genome architecture, sample metadata, and geo-spatial information.

The analysis toward the phylogenetic and genomic sequence of the virus shows a diverse and dynamic picture. After October, emerging viral lineages are seen to be gradually replacing the earlier predominant lineages B.1.1.306 and B.6* (Figure 3A). The lineage B.1.1.7 (alpha variant) was first documented in India in October 2020 followed by B.1.617 and its sub-lineages - B.1.617.2 (delta variant) distinguished by the T478K mutation, and B.1.617.1 (kappa variant) with E484Q spike mutation, from December 2020. Confirmed daily new cases in India had increased from 6 million/ day in September 2020 to 12 million/ day in March 2021 (MOHW, 2021), followed by regional outbreaks in April 2021. We take a closer look at the national trends in viral propagation by digging deeper into the state-wise phylogenetic data. In the state of Maharashtra, after the seeding of B.1.1.7 and B.1.617* in October 2020, there was a rise in cases increasing rapidly from 1.3 million cases daily in September 2020 to 2.8 million cases daily in March 2021 (MOHW, 2021). Similar trends have been observed in other states as well. Results of our study are focused toward understanding the genomic evolution at the phylogenetic level.

SARS-CoV-2, like other RNA viruses, relies on genetic variation for fitness, survival, and, most likely, virulence. A study on the origins of SARS-CoV-2 revealed that random mutations and recombination are the two major sources of genomic variation in this virus (Domingo, 2010). Our analysis shows SARS-CoV-2 to have 9.48e-4 substitutions per site per year or 28.44 nucleotide substitutions per year. Although many efforts have been made to elucidate the behavior of the virus, based on its genetics, there are still some concerns regarding the emerging variants/mutations. Therefore, viral sequencing, data availability, and specific data display are essential to track viral evolution and spread patterns. Other efforts toward building an interactive display of India-specific SARS-CoV-2 genomic data have been observed from India such as https://nextstrain.org/community/banijolly/Phylovis/COVID-India (Jolly and Scaria, 2021) and https://data.ccmb.res.in/gear19/, that use data from GISAID and other genomic surveillance initiatives. To supplement this, our study herein presents an approach towards explaining genomic epidemiology by using globally shared public data from GISAID, building an interactive genomic viewer, and augmenting the observations from similar efforts. This kind of approach toward genomic epidemiology can provide a relatively better view of rising VOC/VOIs in an area, thus enabling better prediction of massive surges in case of positivity rates and thereby facilitating timely healthcare management in the area.

Our study also highlights the importance of data sharing on global platforms such as GISAID. The lower the time gap between the samples collected, sequencing, analysis, and sharing on the global platforms, the better the chances of detecting the VOCs. It also highlights the advantage toward doing longitudinal analysis to capture the trends and significant insights. This is especially true for a country with as big dimensions as India. In hindsight, this is especially true during the delta variant–led second surge in India which stretched the healthcare and medical support system in India. This is also during the phase when there was relatively lower positivity to keep track of the mutations which may be longitudinally changing frequency in a region-wise manner.

A potential limitation of the study could be the uneven distribution of sequencing data and the incomplete information of the clinical outcomes and collection dates of some of the sequences submitted to GISAID. This lack of information led to filtering out of 703 high-quality sequencing data from further analysis. The first case of B.1.617.2 was first seen in states of Bihar and then in Jharkhand, but due to fewer sequence data, the potential spread of this strain could not be followed in detail over the next few months. We are confident that through the INSACOG initiative, this limitation would give away in future to more homogeneous distribution of the sequencing data across the length and breadth of India. It is also important to highlight that sequencing capacity building has happened tremendously which would be augmenting the number of SARS-CoV-2 genomes sequenced, sample to sequence time taken, and global data sharing. Global data sharing also enables analysis of the data by researchers with complementary and unique analysis strengths.

Although a large number of studies have been undertaken toward this avenue, having a clear strain level understanding of the virus has multiple real world applications. This includes strain-targeted spread control measures at key geographical hotspots, thereby holding potential toward easing the burden on public health systems to mitigate loss of life. This knowledge can also be used to understand the characteristics of the virus at an epidemiological level and thus classify specific strains as high- or low-risk categories. This categorization can be performed by observing strain behavior as seen in our study, wherein we see a strain to replace an existing strain at a rapid rate leading to a surge, wherein we can utilize interactive data visualization systems such as Auspice.

## Conclusion

The study highlights the longitudinal Indian trends of SARS-CoV-2 genome evolution between the months of April 2020 to March 2021. It captures the viral genome features in time and space with variability across states and UTs of India. We observed the changing dynamics of Indian predominant lineages of B.1.1.306 and B.6 by newer strains of B.1.1.7 and B.1.617* across the period of 12 months. Based on our analysis, we observe the SARS-CoV-2 mutation rate of 9.48e-4 substitutions per site per year, or 28.44 nucleotide substitutions per year that will result in subsequent evolution of the virus and produce new novel variants.

## Methods

### SARS-CoV-2 Data Retrieval and Curation

In-house sequencing and data analysis of 1144 SARS-CoV-2 genomes were performed using Oxford Nanopore Technology (ONT) and Illumina Miseq platforms, and the pipelines used were the same as detailed in our prior studies (Mehta et al., 2021; Shastri et al., 2021; Kumar et al., 2021). In-house data have also been uploaded to GISAID. Additionally, a total of 9618 SARS-CoV-2 genomic sequences and corresponding patients’ metadata from India reported between April 2020 and March 2021 were retrieved from GISAID (Elbe and Buckland-Merrett, 2017). All the genomic data were combined and the low-quality (>1% missing bases) sequences were filtered out from the final dataset. Genomic sequences which lacked metadata such as date of collection, age, gender, and location information were also removed from the final dataset using the python script on Jupyter Notebook (Jupyter., 2021). The final dataset was of 7631 SARS-CoV-2 genomes.

### Phylogenetic and Evolution Analysis

The Wuhan reference genome for SARS-CoV-2 (NC_045,512.2) was used to perform multiple sequence alignment of 7631 genome sequences of SARS-CoV-2 using Multiple Alignment using Fast Fourier Transform MAFFT v7.475 (Katoh et al., 2002). Subsequently, single nucleotide polymorphisms (SNPs) were extracted from the aligned FASTA sequences using SNP-sites algorithm v2.5.1 (Page et al., 2016), and the resulting variant call format (VCF) file was used for downstream analysis. For clade classification assignment, we used the NextClade command line interface (Hadfield et al., 2018), and the lineage classification was performed with the Phylogenetic Assignment of Named Global Outbreak Lineages (PANGOLIN) using command line (Rambaut et al., 2020).

### Mutation Analysis

Using the vcf file, the top most frequent non-synonymous mutations in the Indian cohort were extracted, and a heatmap was generated using matplotlib (Hunter, 2007) and seaborn (Waskom, 2021) package. The lollipop plot was generated in R using g3viz (Wickham, 2016), rtracklayer (Lawrence et al., 2009), and trackViewer (Ou and Zhu, 2019) packages followed by data visualization using the ggplot2 (Wickham, 2016) package.

### Interactive Phylogeographic Data Visualization

Out of the 7631 sequences, 703 sequences were dropped due to incomplete metadata. Henceforth, 6,928 sequences from the phylogenetic analysis were used to produce a phylogenetic tree, transmission map, mutation spectrum, and genomic divergence using the Nextstrain package Augur, containing python scripts for various filtering steps throughout the Nextstrain’s pipeline (Huddleston et al., 2021). The filter step removes sequences without a collection date, geographical location, and sequences included in the exclude file (Hadfield et al., 2018). The strain names, collection date, latitude, and longitude information are connected to the phylogenetic tree and geographical map with Augur. Some changes were made to the original commands provided by Nextstrain’s SARS-CoV-2 build on Github (GitHub., 2021) to include our samples in the phylogenetic tree. The filter was grouped by the attributes of state, year, and month. The Augur traits command was changed to columns by division, region, and country. The division attribute was added so that the geographical map could display transmission lines by state. To find sequence similarities and differences, MAFFT v7.475 was used (Katoh et al., 2002). To further predict mutations and find the root of phylogenetic branches by establishing traits and common ancestors, a combination of IQTreev2.0.3 and TreeTime v0.8.1 algorithms was used (Sagulenko et al., 2018; Nguyen and Götz, 2016). In the analysis, a tsv file is used to define clades and emerging lineages. For generating an interactive visualization web tool for the obtained phylogeographic data, Auspice (Nextstrain) is used. Auspice is built with Javascript and loads our data from the json file generated by Augur. The web tool is hosted through Indiana University Purdue University (IUPUI) servers at the website (auspice/India). All the figures were edited using Inkscape software (Inkscape., 2021) and BioRender (BioRender., 2021).

## Data Availability

The SARS-CoV-2 genome sequence datasets analysed during this study are publicly available and accessible through the GISAID platform (GISAID - Initiative). The latest Nextstrain Dashboard is available in Supplementary Material.
