# Supplementary material for: Geographical Landscape and Transmission Dynamics of SARS-CoV-2 Variants Across India: A Longitudinal Perspective
Source: Front Genet. 2021 Dec 17;12:753648. doi: 10.3389/fgene.2021.753648 (PMC8719586; doi:10.3389/fgene.2021.753648)
Supplement: Supplementary file 1 [file DataSheet1.ZIP › Supplementary_Longitudinal SARS-CoV-2 landscape of India.docx]

**Geographical landscape and transmission dynamics of SARS-CoV-2 variants across India: a longitudinal perspective**

Neha Jha^1,*^, Dwight Hall^3,*^, Akshay Kanakan^1,*^, Priyanka Mehta^1,*^, Ranjeet Maurya^1, 2^, Quoseena Mir^3^, Hunter Mathias Gill^3^, Sarath Chandra Janga^3,#^, Rajesh Pandey^1, 2,#^

^1^INtegrative GENomics of HOst-PathogEn (INGEN-HOPE) laboratory, CSIR-Institute of Genomics and Integrative Biology (CSIR-IGIB), Mall Road, Delhi-110007, India.

^2^Academy of Scientific and Innovative Research (AcSIR), Ghaziabad-201002, India.

^3^Department of BioHealth Informatics, School of Informatics and Computing, Indiana University Purdue University, Informatics and Communications Technology Complex, IT475H, 535 West Michigan Street, Indianapolis, IN, 46202, USA.

^*^ Joint First-authors

^#^ Co-corresponding Authors

For correspondence:

**Rajesh Pandey, PhD**

Principal Scientist,

INtegrative GENomics of HOst-PathogEn (INGEN-HOPE) laboratory,

CSIR-Institute of Genomics and Integrative Biology (CSIR-IGIB),

Associate Professor, Academy of Scientific & Innovative Research (AcSIR),

North Campus, Mall Road, Delhi-110007, India.

https://www.igib.res.in/?q=RajeshPandey

Contact: rajeshp@igib.in; Tel.: +91 9811029551

Or

**Sarath Chandra Janga, PhD**

Associate Professor, School of Informatics and Computing

Indiana University (IUPUI)

719 Indiana Avenue, Room 319, Walker Plaza Building

Indianapolis, Indiana – 46202

Contact: scjanga@iupui.edu

Tel: +1-317-278-4147, Fax: +1-317-278-9201

**Supplementary information**

**Supplementary Table 1**: WHO classification of SARS-CoV-2 as Variants Of Concern (VOC) and Variants Of Interest (VOI) as of 15 June 2021.

| **Variants of concern** | | | | | | |
| --- | --- | --- | --- | --- | --- | --- |
| **WHO label** | **Pango**  **lineage** | **Nextstrain**  **clade** | **Earliest documented**  **samples** | **Key Mutations** | **Strain properties** | **References** |
| Alpha | B.1.1.7 | 20I/S:501Y.V1 | United Kingdom,  Sep-2020 | S:N501Y, S:S69-, | Increased transmissibility, Increased Severity | (Davies et al., 2021a, 2021b) |
| Beta | B.1.351 | 20H/S:501Y.V2 | South Africa,  May-2020 | S:K417N, S:E484K | Increased transmissibility, Increased Severity | (Tegally et al., 2021; Funk et al., 2021) |
| Gamma | P.1 | 20J/S:501Y.V3 | Brazil,  Nov-2020 | S:N501Y, S:E484K, S:K417T, | Increased Transmission, Immune evasion | (Faria et al., 2021; de Souza et al., 2021) |
| Delta | B.1.617.2 | 21A/S:478K | India,  Oct-2020 | S:S478K, S:L452R, S:T78K | Increased Transmission, Immune evasion | (Dhar et al., 2021; Wall et al., 2021) |
| **Variants of interest** | | | | | | |
| **WHO label** | **Pango**  **lineage** | **Nextstrain**  **clade** | **Earliest documented**  **samples** | **Key Mutations** | **Strain properties** | **References** |
| Epsilon | B.1.427/B.1.429 | 20C/S.452R | United States of America,  Mar-2020 | S:L452R | Increased Transmission, Immune evasion | (Zhang et al., 2021; McCallum et al., 2021) |
| Zeta | P.2 | 20B/S.484K | Brazil,  Apr-2020 | S:E484K | Immune evasion | (Sabino et al., 2021; Greaney et al., 2021) |
| Eta | B.1.525 | 20A/S484K | Multiple countries,  Dec-2020 | S:T19R, S:R158G, S:T478K | Immune evasion | (Suryadevara et al., 2021) |
| Theta | P.3 | 20B/S:265C | Philippines,  Jan-2021 | E1092K, H1101Y, V1176F | Immune evasion | (Jangra et al., 2021) |
| Iota | B.1.526 | 20C/S:484K | United States of America,  Nov-2020 | S:L5F, S:T95I, S:D253G | Increased Transmission, Immune evasion | (Annavajhala et al., 2021) |
| Kappa | B.1.617.1 | 21A/S:154K | India,  Oct-2020 | S:E154K, S:E484Q, S:Q1071H | Immune evasion | (Ferreira et al., 2021) |

**Supplementary Figure 1:** Frequency distribution of lineages with respect to time in Bihar.

**
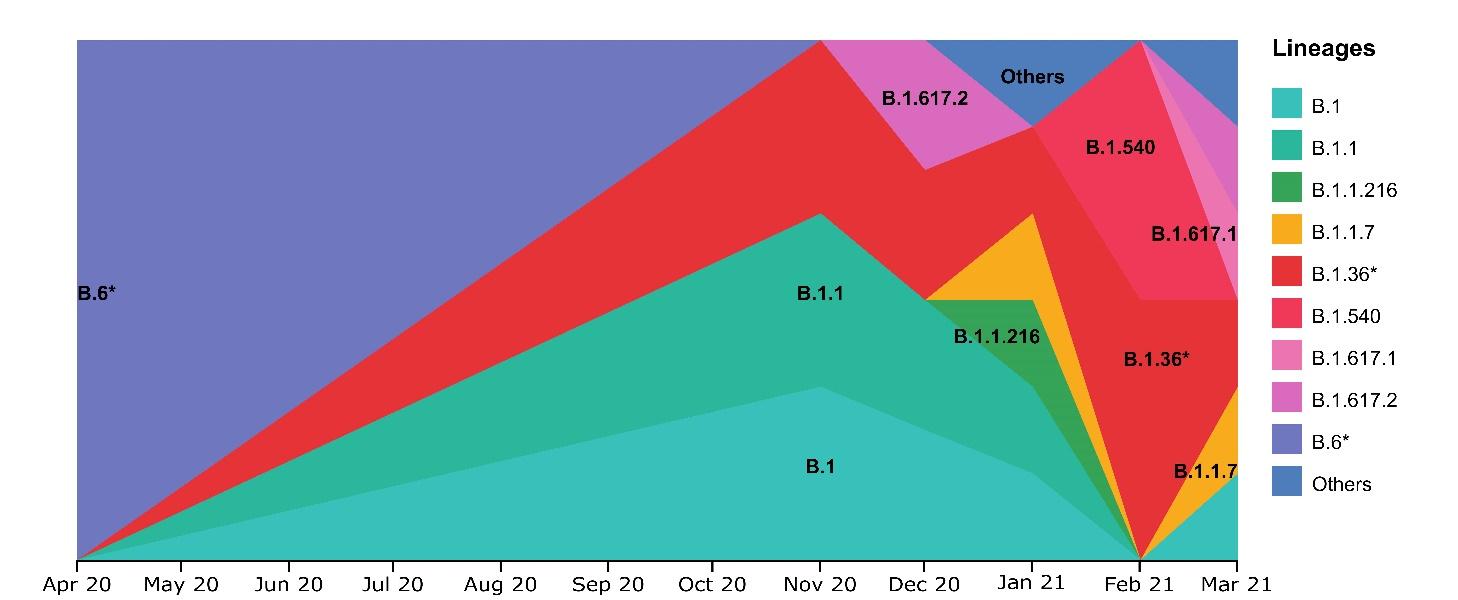
**

**Supplementary Link 1: Updated Nextstrain Dashboard From April-2020 to September-2021**

**<https://covid19-indiana.soic.iupui.edu/India/EmergingLineages/up/to/Sept2021>**

**Bibliography**

Annavajhala, M. K., Mohri, H., Zucker, J. E., Sheng, Z., Wang, P., Gomez-Simmonds, A., Ho, D. D., and Uhlemann, A.-C. (2021). A Novel SARS-CoV-2 Variant of Concern, B.1.526, Identified in New York. *medRxiv*. doi:10.1101/2021.02.23.21252259.

Davies, N. G., Abbott, S., Barnard, R. C., Jarvis, C. I., Kucharski, A. J., Munday, J. D., Pearson, C. A. B., Russell, T. W., Tully, D. C., Washburne, A. D., et al. (2021a). Estimated transmissibility and impact of SARS-CoV-2 lineage B.1.1.7 in England. *Science* 372. doi:10.1126/science.abg3055.

Davies, N. G., Jarvis, C. I., CMMID COVID-19 Working Group, Edmunds, W. J., Jewell, N. P., Diaz-Ordaz, K., and Keogh, R. H. (2021b). Increased mortality in community-tested cases of SARS-CoV-2 lineage B.1.1.7. *Nature* 593, 270–274. doi:10.1038/s41586-021-03426-1.

de Souza, W. M., Amorim, M. R., Sesti-Costa, R., Coimbra, L. D., Toledo-Teixeira, D. A. de, Parise, P. L., Barbosa, P. P., Bispo-dos-Santos, K., Mofatto, L. S., Simeoni, C. L., et al. (2021). Levels of SARS-CoV-2 Lineage P.1 Neutralization by Antibodies Elicited after Natural Infection and Vaccination. *SSRN Journal*. doi:10.2139/ssrn.3793486.

Dhar, M. S., Marwal, R., Vs, R., Ponnusamy, K., Jolly, B., Bhoyar, R. C., Fatihi, S., Datta, M., Singh, P., Sharma, U., et al. (2021). Genomic characterization and Epidemiology of an emerging SARS-CoV-2 variant in Delhi, India. *medRxiv*. doi:10.1101/2021.06.02.21258076.

Faria, N. R., Mellan, T. A., Whittaker, C., Claro, I. M., Candido, D. da S., Mishra, S., Crispim, M. A. E., Sales, F. C. S., Hawryluk, I., McCrone, J. T., et al. (2021). Genomics and epidemiology of the P.1 SARS-CoV-2 lineage in Manaus, Brazil. *Science* 372, 815–821. doi:10.1126/science.abh2644.

Ferreira, I., Datir, R., Papa, G., Kemp, S., Meng, B., Rakshit, P., Singh, S., Pandey, R., Ponnusamy, K., Radhakrishnan, V. S., et al. (2021). SARS-CoV-2 B.1.617 emergence and sensitivity to vaccine-elicited antibodies. *BioRxiv*. doi:10.1101/2021.05.08.443253.

Funk, T., Pharris, A., Spiteri, G., Bundle, N., Melidou, A., Carr, M., Gonzalez, G., Garcia-Leon, A., Crispie, F., O’Connor, L., et al. (2021). Characteristics of SARS-CoV-2 variants of concern B.1.1.7, B.1.351 or P.1: data from seven EU/EEA countries, weeks 38/2020 to 10/2021. *Euro Surveill.* 26. doi:10.2807/1560-7917.ES.2021.26.16.2100348.

Greaney, A. J., Loes, A. N., Crawford, K. H. D., Starr, T. N., Malone, K. D., Chu, H. Y., and Bloom, J. D. (2021). Comprehensive mapping of mutations in the SARS-CoV-2 receptor-binding domain that affect recognition by polyclonal human plasma antibodies. *Cell Host Microbe* 29, 463–476.e6. doi:10.1016/j.chom.2021.02.003.

Jangra, S., Ye, C., Rathnasinghe, R., Stadlbauer, D., Personalized Virology Initiative study group, Krammer, F., Simon, V., Martinez-Sobrido, L., García-Sastre, A., and Schotsaert, M. (2021). SARS-CoV-2 spike E484K mutation reduces antibody neutralisation. *Lancet Microbe* 2, e283–e284. doi:10.1016/S2666-5247(21)00068-9.

McCallum, M., Bassi, J., De Marco, A., Chen, A., Walls, A. C., Di Iulio, J., Tortorici, M. A., Navarro, M.-J., Silacci-Fregni, C., Saliba, C., et al. (2021). SARS-CoV-2 immune evasion by the B.1.427/B.1.429 variant of concern. *Science*, eabi7994. doi:10.1126/science.abi7994.

Sabino, E. C., Buss, L. F., Carvalho, M. P. S., Prete, C. A., Crispim, M. A. E., Fraiji, N. A., Pereira, R. H. M., Parag, K. V., da Silva Peixoto, P., Kraemer, M. U. G., et al. (2021). Resurgence of COVID-19 in Manaus, Brazil, despite high seroprevalence. *Lancet* 397, 452–455. doi:10.1016/S0140-6736(21)00183-5.

Suryadevara, N., Shrihari, S., Gilchuk, P., VanBlargan, L. A., Binshtein, E., Zost, S. J., Nargi, R. S., Sutton, R. E., Winkler, E. S., Chen, E. C., et al. (2021). Neutralizing and protective human monoclonal antibodies recognizing the N-terminal domain of the SARS-CoV-2 spike protein. *Cell* 184, 2316–2331.e15. doi:10.1016/j.cell.2021.03.029.

Tegally, H., Wilkinson, E., Giovanetti, M., Iranzadeh, A., Fonseca, V., Giandhari, J., Doolabh, D., Pillay, S., San, E. J., Msomi, N., et al. (2021). Detection of a SARS-CoV-2 variant of concern in South Africa. *Nature* 592, 438–443. doi:10.1038/s41586-021-03402-9.

Wall, E. C., Wu, M., Harvey, R., Kelly, G., Warchal, S., Sawyer, C., Daniels, R., Hobson, P., Hatipoglu, E., Ngai, Y., et al. (2021). Neutralising antibody activity against SARS-CoV-2 VOCs B.1.617.2 and B.1.351 by BNT162b2 vaccination. *Lancet* 397, 2331–2333. doi:10.1016/S0140-6736(21)01290-3.

Zhang, W., Davis, B. D., Chen, S. S., Sincuir Martinez, J. M., Plummer, J. T., and Vail, E. (2021). Emergence of a Novel SARS-CoV-2 Variant in Southern California. *JAMA* 325, 1324–1326. doi:10.1001/jama.2021.1612.
